# Experimental validation of new self-voltage balanced 9L-ANPC inverter for photovoltaic applications

**DOI:** 10.1038/s41598-021-84531-z

**Published:** 2021-03-03

**Authors:** M. Jagabar Sathik, Dhafer J. Almakhles, N. Sandeep, Marif Daula Siddique

**Affiliations:** 1 Renewable Energy Lab, College of Engineering, Prince Sultan University, Riyadh, Saudi Arabia; 2 SRM Institute of Science and Technology, Kattankulathur Campus, Chennai, India; 3 Malviya National Institute of Science and Technology, Jaipur, India; 4 Univerity of Malaya, Kuala Lumpur, Malaysia

**Keywords:** Electrical and electronic engineering, Photovoltaics

## Abstract

Multilevel inverters play an important role in extracting the power from renewable energy resources and delivering the output voltage with high quality to the load. This paper proposes a new single-stage switched capacitor nine-level inverter, which comprises an improved T-type inverter, auxiliary switch, and switched cell unit. The proposed topology effectively reduces the DC-link capacitor voltage and exhibits superior performance over recently switched-capacitor inverter topologies in terms of the number of power components and blocking voltage of the switches. A level-shifted multilevel pulse width modulation scheme with a modified triangular carrier wave is implemented to produce a high-quality stepped output voltage waveform with low switching frequency. The proposed nine-level inverter’s effectiveness, driven by the recommended modulation technique, is experimentally verified under varying load conditions. The power loss and efficiency for the proposed nine-level inverter are thoroughly discussed with different loads.

## Introduction

In recent decades, the modernization of individuals and the development of renewable energy technology are increasing worldwide, driven by the alert on global warming. To reduce CO_2_ emission and generate more electricity to meet demand, the solar energy system is an important option capable of generating power, ranging from a few watts to megawatts. In order to generate high power in terms of megawatts ranges, the power electronics converters play a major role. In this, multilevel inverters (MLIs) are predominant power converters which are highly suitable for medium and high voltage applications like high power AC drives, FACTS devices, HVDC transmission, and large-scale wind and photovoltaic systems^1,2^. Compared to the traditional two-level inverter, the MLIs have low voltage stress on power devices, low electromagnetic interference, low total harmonic distortion (THD), reduced common-mode voltage, and enhanced output voltage (V_out_)^3,4^. The first MLI reported in 1975 is by Baker and was named as cascaded H-bridge inverter (CHB). Next, both neutral point clamp (NPC) and flying capacitor, referred to as a floating capacitor (FC) topology were introduced in 1980–1981 by A. Nabae.

Generally speaking, the conventional MLI topologies have been known for their good modularity and low voltage stress on switches. Nevertheless, they have a higher number of switching devices, clamping diodes, isolated dc sources, and bulky dc-link capacitors^5^. In addition, they are only suitable for constant isolated dc source. In other words, the conventional MLIs are not suitable for photovoltaic (PV) applications since the output voltage of PV fluctuates due to the uncertainty of the solar irradiance and temperature. Further, the output voltage of PV is relatively low, in which the dc/dc boost converter is used on the front side of the inverter to regulate and boost the PV output voltage. However, as the output voltage is boosted at a higher level than the desired, the input voltage (V_in_) is boosted to match the load requirement, which gives more burden in terms of high voltage stress and high value of the magnetic component to the front-end dc/dc converter. To reduce the front-end dc/dc converter voltage rating, a various switched capacitor MLI is proposed with voltage gain not less than one. In particular, the neutral point-based topologies need a higher input voltage, which can be rectified using the boost type inverter.

The combination of conventional topologies and other recently developed topologies forms the so-called hybrid MLI.

For example, the active neutral point clamped (ANPC) inverter topology is widely used for induction motor drive or AC grid-connected applications. The output voltage of the ANPC topology is half of the input voltage, and hence, it needs a high dc-link capacitor. Among the various hybrid ANPC topologies, the combination of ANPC and FC has gained more attention due to a single dc source generating a higher number of voltage levels. The switched capacitor MLI (SCMLI) topologies, which are capable of generating a 9L output voltage waveform, are widely presented in^6–14^. A new switched capacitor topology without the capacitor voltage sensor is proposed in^6^. Voltage balancing of the capacitors is achieved using a logic function, and it is embedded into a pulse decoder. In^7^, both the high and low voltage switches are operated by low and high switching frequencies, respectively, to reduce the power losses, and the capacitors are naturally balanced. A 9L double hybrid active NPC inverter topology presented in^8^ employs digital logic functions to balance capacitor voltage with the help of a voltage sensor. However, these topologies output voltage is equal to the input voltage.

To resolve the above problems, SCMLIs with boosting ability topologies are introduced in^9–14^. In^9^, a multicell structure with self-capacitor voltage balancing and boosting is presented. The full-bridge inverter circuit is used to produce an alternate output voltage waveform. However, the number of switches and voltage ratings of switches are considerably high. A new 9L quadratic boost converter topology with the self-voltage balancing is proposed in^10,11^. Nevertheless, it needs a capacitor and switches with a diversified voltage rating. Further, the voltage rating of the capacitor is higher than the input voltage, which limits its application to high voltage fields. A single-stage compact MLI with self-voltage balancing and boosting is presented in^12–14^. Here, the number of power components and blocking voltage on the switches are reduced. The voltage rating of the capacitor is half of the input voltage in^12,13^, but in^14^, the rating of the capacitor is equal to the input voltage. However, the previously mentioned topologies are not optimized concerning a lower number of power components or voltage stress on switches. Further, the voltage rating of the capacitor is also higher in a few topologies, which motivates this study to present a novel voltage boosting type topology with reduced switch count.

This paper successfully develops a 9L-ANPC topology to overcome the drawbacks of the existing topologies with low voltage rating components devised. The pertinent advantages of the proposed topology are

1. The conventional NPC and ANPC topologies output voltage is half of the input voltage, which is rectified, and the output voltage is boosted to be equal to the input voltage.
2. It does not require a sensor to measure the capacitor voltage.
3. In the front end, the low voltage dc/dc boost converters are required.
4. Due to less number of components, it features reduced power losses.
5. The capacitor voltages are independent of the load power factor.
6. The proposed modulation scheme offers low THD.

A detailed discussion about the proposed structure, modes of operation, loss calculation, and experimental validations are presented in the sequel.

## Proposed 9L-ANPC inverter

**Figure 1:**
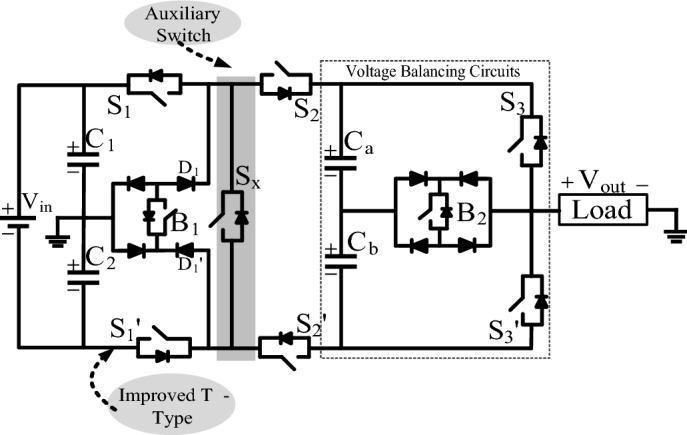
Proposed single-phase basic unit for 9L operation.

The proposed 9L switched-capacitor inverter topology is shown in Fig. 1. It comprises an improved T-type inverter, auxiliary switch, and switched cell (SC) unit. The improved T-type inverter consists of two dc-link capacitors (C_1_, C_2_) connected in parallel with the input dc source (V_in_), two unidirectional IGBT’s with anti-parallel diodes (S_1_, S_1_′), and one bidirectional switch (B_1_). The SC consists of two series-connected capacitors (C_a_ and C_b_), four unidirectional IGBT’s with anti-parallel diodes (S_2_, S_2_′, S_3_, and S_3_′), and one bidirectional switch (B_2_). The Auxiliary switch (S_x_) is used to discharge the capacitors C_a_ and C_b_ during positive and negative half-cycles. The input voltage is shared among the dc-link capacitors C_1_ and C_2_, in which V_C1_ = V_C2_ = V_in_/2 and V_o_ = M × V_in_ (M-modulation index, V_o_ = V_out_), balanced by the switch B_1_.

The capacitors C_1_ and C_2_ supply the dc voltage to the load during positive and negative half-cycle, respectively. The fundamental operation of proposed 9L inverter is given in Table 1.

**Table 1:** Fundamental operation of proposed 9L inverter.

Voltage balancing	Voltage boosting
Since only two capacitors are connected in series with mid-point neutral connection. In zero state the switches B_1_ is ON and provide the zero potential at point ‘N’ point Switched cell capacitors are rated to V_in_/4 In charging mode, the dc-link capacitor C_1_ and C_2_ is connected in parallel with C_a_ and C_b_ In discharging mode, the dc-link capacitor C_1_ and C_2_ is connected in series with C_a_ and C_b_	The auxiliary switch is used to connect the dc-link and FC in series The ratio of input to the output voltage is 1:1 in proposed 9L inverter During the parallel connection dc-link capacitor and FC, the FC is charging the voltage to V_in_/4

**Figure 2:**
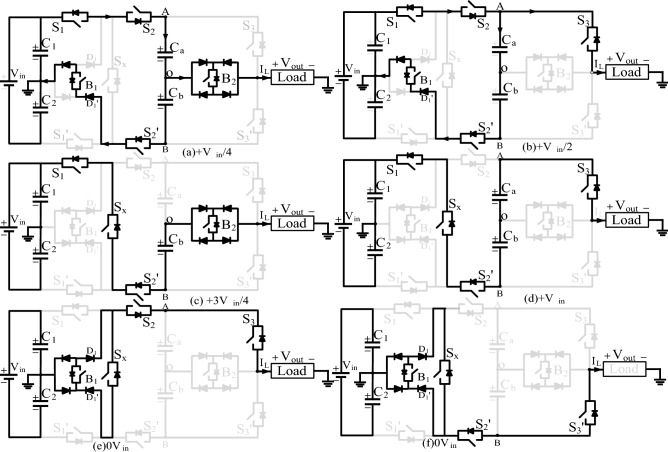
Proposed ANPC 9L-BSCI operation (**a**–**f**) modes of operation of the positive half cycle.

As shown in Fig. 2, the designated points of output A, O, and B are the node points used to calculate the RMS voltage. Initially, at point A, V_in_/2 is present, i.e. (V_in_/2 − 0), and the voltage difference between point O to A is V_in_/2 − V_Ca_ = V_in_/4. At this point, the switch B_2_ is turned on. At point B, the capacitors are charged to V_in_/4 and the voltage, i.e., V_in_/2 − V_Ca_ − V_Cb_ = 0 V. The current path for each voltage level and the corresponding switching sequence are given in Table 1, and for simple understanding, only the positive half-cycle and zero states are depicted in Fig. 2a–f. Mode 1 (+ V_in_/4): In-state 1 and 2, the capacitors C_a,_ and C_b_ are charged to V_in_/4 through switches S_1_, S_2_, S_2_′, D′ and B_1_, simultaneously the switch B_2_ is turned ON to produce output voltage equals to V_in_/4, as given in Fig. 2a. Mode 2 (+ V_in_/2): The capacitors C_a,_ and C_b_ charge through switches S_1_, S_2_, S_2_′, D′ and B_1_, simultaneously the switch S_3_ is turned ON to produce output voltage equals to V_in_/2 as given in Fig. 2b. Mode 3 (+ 3V_in_/4): States 3 and 4 discharge the capacitors C_a_ and C_b_. To obtain + 3V_in_/4 at the load, the capacitor C_1_ is connected to point B through auxiliary switch S_x_. The load current (I_L_) flows through S_1_, S_x_, S_2_′ and B_1_ to produce a third voltage level (+ 3V_in_/4), as shown in Fig. 2c. Mode 4 (+ V_in_): Both the switched cell capacitors are discharged through switches S_1_, S_x_, S_2_′ and S_3_ as shown in Fig. 2d. Hence, the negative half-cycle is obtained by choosing the corresponding switching path. During state 3 (± 3V_in_/4), capacitor C_b_ discharges during the positive half-cycle and C_a_ discharges during the negative half cycle. Mode 0 (0 V): The zero states are more essential to provide a freewheeling path to the load current when the load is inductive. The zero states are achieved either by turning on switches B_1_, S_2_, S_3_, D, D′ and S_x_ or B_1_, D, D′, S_x_, S_2_′ and S_3_′ as shown in Fig. 2e,f. The proposed topology consists of two dc-link capacitors and two series-connected FCs. The FC voltages should be maintained to V_in_/4, but the dc-link capacitor voltages are V_in_/2. The output voltage (V_out_) is obtained by using the switching functions, DC-link, and FC using Eq. (1):

1 $$V_{out} = \left( {S_{1} + S_{1}^{\prime } } \right)V_{in} - \left( {S_{2} - S_{2}^{\prime } } \right)V_{Cb} + \left[ {\left( {S_{3} - S_{3}^{\prime } } \right)S_{x} } \right]V_{Ca} - \left( {S_{x} + B_{1} } \right)\;\left( {D^{\prime } - D} \right)V_{Cb} {-}\left( {D^{\prime } - D} \right)V_{Ca} - B_{2} V_{Cb} - S_{1}^{\prime } V_{in} - S_{3}^{\prime } V_{Ca} - S_{x} \cdot S_{1}^{\prime } V_{C2} ,$$

where the V_C2_, V_Ca_ and V_Cb_ are the voltages of dc-link capacitor C_2_, floating capacitor C_a_ and C_b_, respectively. The voltage across the dc-link capacitor and FCs are given in Eq. (2)

2 $$V_{C1} = V_{C2} = \frac{{V_{in} }}{2}, \, V_{Ca} = \, V_{Cb} = \frac{{V_{in} }}{4},$$

The {1,0} is the logic values of the switching function, i.e., the switch is ON state it represents as “1” and for OFF state represent as “0”, respectively. The corresponding switches are turned ON and OFF based on the given switching sequence in Table 2. The capacitance value of the FC depends on the ratio of charging and discharging time. Here, the FCs are charging and discharging is two times, but the duty cycle of charging is higher than the discharging, which means the proposed topology uses a lower number of dc capacitance. However, the current ratings are identical in all the switches. The maximum blocking voltage (MBV) on individual switches is obtained from Eqs. (3)–(5)

3 $$MBV_{S1,S2} = V_{in} ,$$

4 $$MBV_{S2,S2^{\prime}, \, S3,S3^{\prime},B1,Sx} = V_{in} /2,$$

5 $$MBV_{B2} = V_{in} /4,$$

**Table 2:** Switching sequence of each level.

Mode	On-state switches	Output voltage (V_out_)	Capacitors	
			V_Ca_	V_Cb_
1	S_1_, S_2_, B_1_, B_2,_ S_2_′, D’/S_2_, S_2_′, S_1_′, B_1_, B_2_	+ V_in_/4/− V_in_/4	C	
2	S_1_, S_2_, B_1_, S_3,_ S_2_′, D’/S_2_, S_3_′, S_2_′, S_1_′, B_1_	+ V_in_/2/− V_in_/2	C	
3	S_1_, S_x_, S_2_′, B_2,_/S_2_, S_x_, S_1_′, B_2_	+ 3V_in_/4/− 3V_in_/4	–	D
			D	–
4	S_1_, S_x_, S_2_′, S_3_/S_2_, S_x_, S_3_′, S_1_′	+ V_in_/− V_in_	D	D
0	B_1_, D′, D, S_2_, S_3_,/B_1_, D’, D, S_2_′, S_3_′	0V_in_	–	–

*C* charging, *D* discharging, – no effect.

Total blocking voltage (TBV) is the sum of the blocking voltages on individual switches specified in per unit value, given in Eqs. (6)–(8).

6 $$TBV_{p \cdot u} = MBV_{S1,S1^{\prime}} + \, MBV_{S3,S3^{\prime},S2,S2^{\prime},B1,Sx} + \, MBV_{B2} ,$$

7 $$TBV_{p \cdot u} = 2V_{in} + 3V_{in} + 0.5V_{in} ,$$

8 $$TBV_{p \cdot u} = 5.5V_{in} .$$

## Modified multicarrier triangular carrier signal

**Figure 3:**
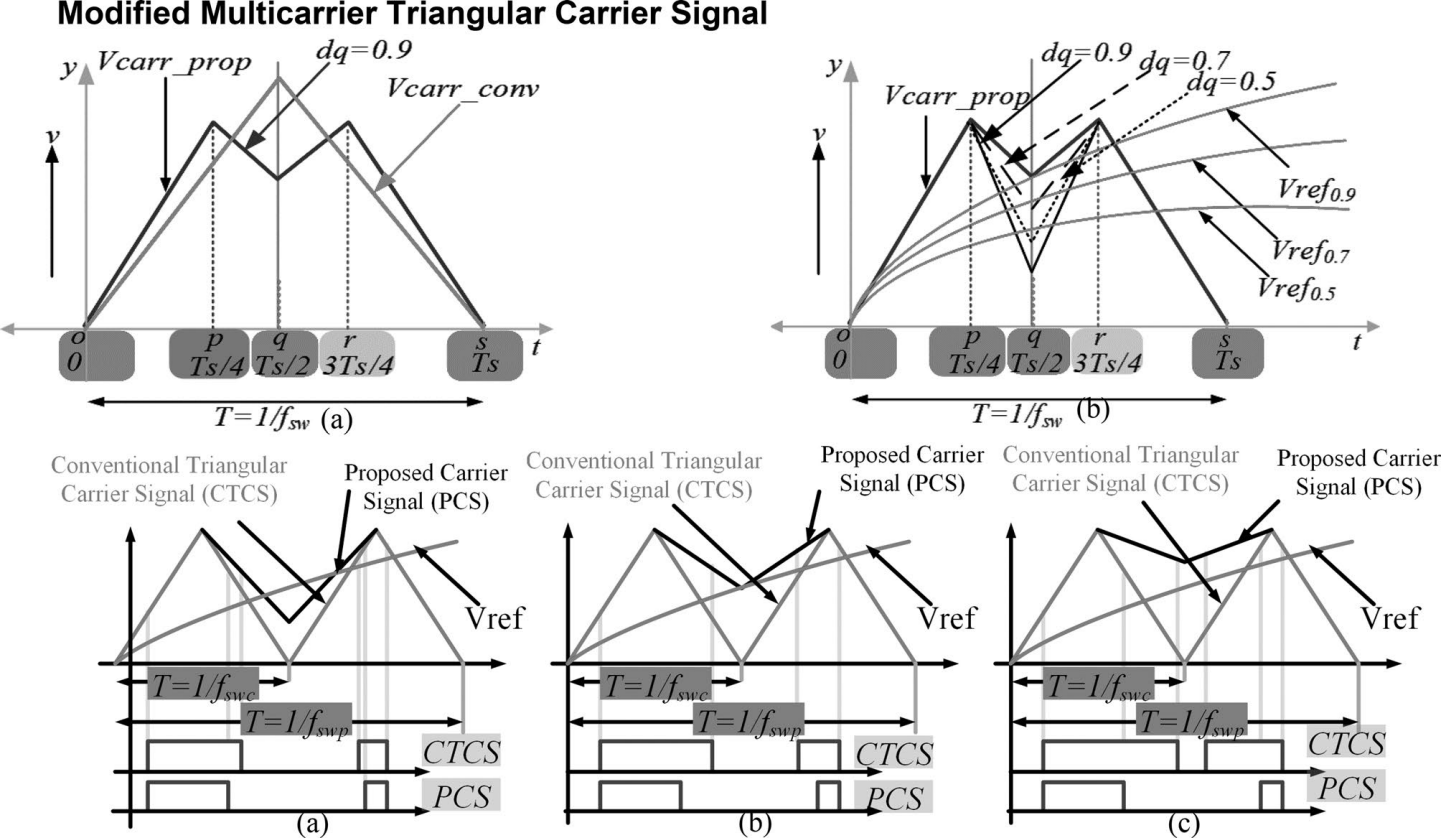
Proposed new carrier signal waveform (**a**) compared with conventional triangular waveform (**b**) with various *dq* points (**c**) comparison of proposed and triangular carrier signal with different pulse width variations.

The conventional multicarrier PWM technique is used for low THD when the switching frequency is high. In order to reduce the switching frequency with reduced THD and high voltage RMS, a multicarrier level-shifted modulation scheme is introduced. The conventional carrier waveform under level-shifted multilevel pulse width modulation is decomposed into two intervals ranging from 0 to Ts/2 and Ts/2 to Ts with the amplitude of voltage varying from 0 to 1 and from 1 to 0 respectively in the mentioned time intervals as shown in Fig. 3a. To reduce the THDs and to increase the RMS value of the output voltage of the inverter, the proposed switching scheme under level-shifted multilevel pulse width modulation is subjected to a change in the amplitude of the carrier wave with the sampling time period of Ts equally divided into five intervals comprising of 0 to Ts/4, Ts/4 to Ts/2, Ts/2 to 3Ts/4 and 3Ts/4 to Ts. The amplitude in these intervals varies from 0 to 1, 1 to change in q (dq), dq to 1, and from 1 to 0, respectively, as illustrated in Fig. 3b. When dq = 0, the proposed scheme takes the shape of the conventional carrier waveform. When dq = 1, the proposed scheme modifies into the shape of an isosceles trapezoid. When dq = 1, a single pulse is generated in the time interval of Ts. This can further deteriorate the THD. Therefore, to mitigate this detrimental effect on THD, choosing the value of dq is of great significance. Figure 3c clearly shows the difference between the pulse width duration of the proposed carrier signal and the conventional triangular carrier signal.

**Figure 4:**
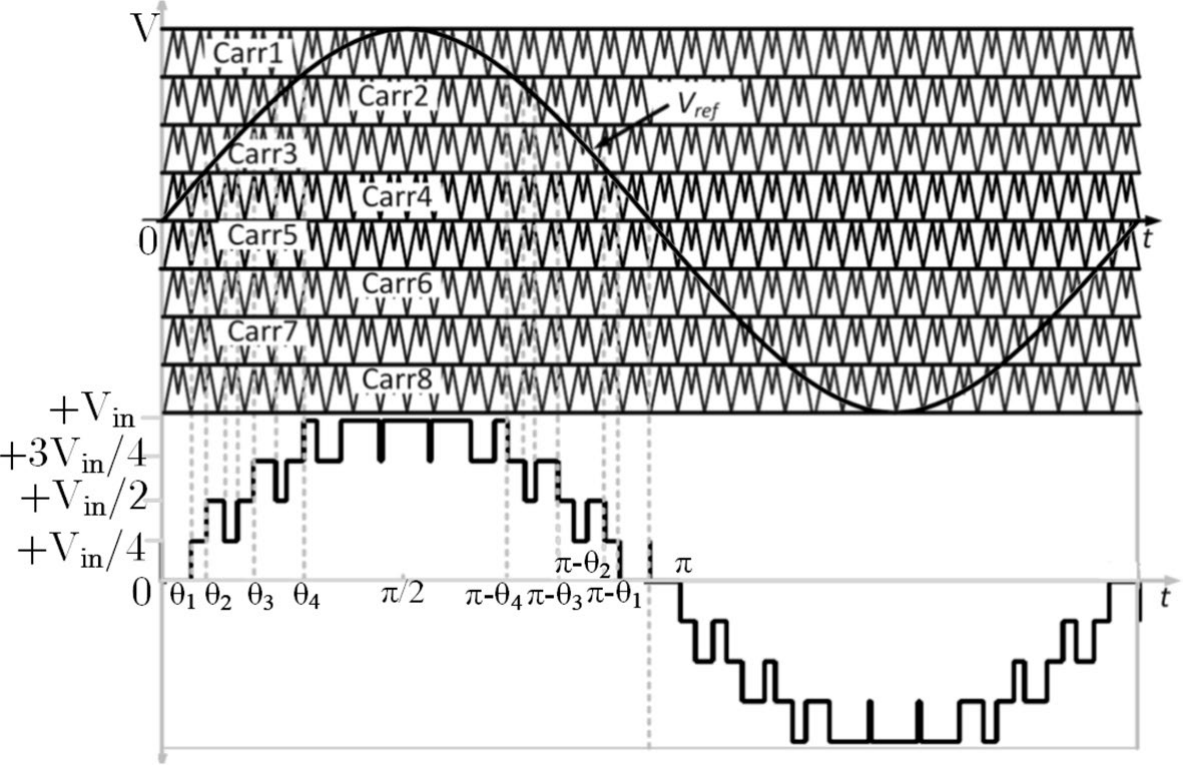
(**a**) Proposed multicarrier signal. (**b**) Typical 9L output voltage waveform.

The optimal value of dq is chosen to be greater than 0 and lesser than 1. The generalized level-shifted carrier signal (V_carr_) with sinusoidal reference signal (V_Ref_) is represented in Fig. 4a and the typical 9L output voltage waveform is shown in Fig. 4b. The Eq. (9) gives the function f(x,y) of two level full bridge inverter is

9 $$f(x,y) = \frac{{A_{00} }}{2} + \sum\nolimits_{{{\text{n}} = 1}}^{\infty } {\left[ {A_{0n} \cos ny + {\text{B}}_{0n} \sin ny} \right]} + \sum\nolimits_{{{\text{m}} = 1}}^{\infty } {\left[ {A_{0m} \cos my + {\text{B}}_{0m} \sin my} \right]} + \sum\nolimits_{m = 1}^{\infty } {\sum\nolimits_{{{\text{m}} = - \infty }}^{\infty } {\left[ {A_{mn} \cos (mx + ny) + {\text{B}}_{mn} \sin (mx + ny)} \right]\quad (n \ne 0),} }$$

where *m* is the carrier index variable and *n* is the baseband index variable. The above equation consists of the fundamental component, and harmonics^15^. Since the proposed topology produces the double pulse when dq ≠ 1, and the Fourier equation can be further reduced and given in Eq. (10)

10 $$A_{00} + jB_{00} = \frac{{2V_{in} }}{{2\pi^{2} }} \times \int\limits_{ - \pi }^{\pi } {\left( {1 + \pi M\cos y} \right) \, dy} ,$$

where ‘M’ is modulation index. Since, the duty ratio of the proposed modulation scheme is higher than the conventional PWM, the proposed topology conduction time is high. Further as the switching angle of each pulses is different from that of the conventional PWM, leads to reduction of THD in proposed PWM technique as shown in Fig. 3c.

## Determination of floating capacitors

The long discharging cycle (LDC) occurs for both the FCs during time interval (*θ*_3_* − (π − θ*_3_*))*. The maximum charge, required by the capacitor is given in (11) where, *i*_*L*_ represents the load current. As *θ*_3_ is obtained as in (12). Similarly, *θ*_4_ can be obtained.

11 $$\Delta Q = \int\limits_{{\theta_{3} }}^{{\pi - \theta_{3} }} {\frac{{i_{L} }}{\omega }} \, d\omega t,$$

12 $$\theta_{3} = \frac{{\sin^{ - 1} \left( {{\raise0.7ex\hbox{${3V_{carr} }$} \!\mathord{\left/ {\vphantom {{3V_{carr} } {V_{ref} }}}\right.\kern-\nulldelimiterspace} \!\lower0.7ex\hbox{${V_{ref} }$}}} \right)}}{{2\pi f_{f} }}.$$

The maximum voltage ripple occurred at the resistive load. So, it is worth mentioning that the maximum discharge value for pure resistive load. Therefore, the *ΔQ* can be calculated as in (9) for capacitor *C*_*a*_ and *C*_*b*_.

13 $$\Delta Q_{C1} = \frac{{v_{in} }}{{2\pi f_{f} R_{L} }} \, \left( {(\pi - \theta_{3} ) - \theta_{3} } \right).$$

The ripple value (*ΔV*_*rip*_) across the capacitor *C*_*a*_ and *C*_*b*_ is obtained (11) as *R*_*L*_ is the resistive load and *f*_*f*_ is the inverter output voltage frequency. The optimum value for each capacitor (*C*_*opt*_) can be given as in (12).

14 $$\Delta V_{rip} = \frac{{v_{in} }}{{2\pi f_{f} R_{L} C}} \times (\pi - 2\theta_{3} ),$$

15 $$C_{opt} = \frac{{v_{in} }}{{2\pi f_{f} R_{L} \Delta V_{rip} }} \times \left( {\pi - 2\theta_{3} } \right).$$

## Comparison of proposed multilevel inverter with other recent MLI topologies

A comparison of different switched capacitor MLI topology and conventional topologies are considered. In order to generate the 9L output voltage at the load, the CHB, NPC, and FC topologies use 16 switches; other than these, the NPC and FC need more clamping diodes, clamping capacitors, and additional dc-link balancing circuits. However, in the case of CHB, it needs four isolated dc sources and no voltage boosting. Apart from the conventional topologies,^10^ developed a topology where eight switches and three diodes are used, but it requires four maximum blocking voltage (MBV) switches. The ratio of the input voltage versus the maximum blocking voltage is 1:4 (V_in_: MBV). Although the topologies in^6,8^ are close to the proposed topology with the total standing voltage of 6 V_in_, these topologies do not have voltage boosting ability, unlike the proposed topology. The other parameters such as voltage rating of the capacitor (V_C,rating_), the number of the capacitors (N_Capacitors_), number of the diodes (N_diode_) and number of sources (N_source_) are compared and presented in Table 3 with recent 9L SCMLI topologies. Further, the recent topologies presented in^16,17^ are compared with the proposed topology. In^16^, the topology does not have boosting ability and it is not an NPC type topology but the^17^ is family of ANPC with high voltage gain with more number of switches. Further, the maximum blocking voltage is equal to the V_out_. However, from the Table 3, its confirming that the proposed topology is superior to the all-other topologies presented in the literature in terms of switch count.

**Table 3:** Comparison of proposed 9L-inverter with conventional and other recent SCMLI topologies.

Topologies	N_Switches_	N_Source_	N_Capacitors_	V_C,rating_	N_Diode_	MBV _Switches_	TBV_p.u_	Additional capacitor balancing	Voltage boosting ability	Efficiency η%	Negative level
NPC	16	1	8	V_in_/4	56	16	4 V_in_	Required	No	NA	Mid-point
FC	16		31	V_in_/4	–	16	4 V_in_	Not required	No	NA	Mid-point
CHB	16	4	–	–	–	16	4 V_in_	–	No		H-Bridge
								Not required		NA	H-bridge
^6^	10	1	3	V_in/_2	–	4	6 V_in_	Required	No	92.3%	Inherent
^7^	12		4	V_in/_2	–	4	10 V_in_	Required	No	98.3%	Inherent
^8^	12		3	V_in/_2	–	2	6 V_in_	Required	No	NA	Mid-point
^9^	9		2	2V_in_	1	4	24 V_in_	Self-balancing	Yes		H-bridge
^10^	8		2	2V_in_	3	2	14 V_in_			> 93%	H-bridge
^11^	12		3	2 V_in_	–	2	24 V_in_			97.3%	Inherent
^13^	12		2	V_in_/2	1	10	12 V_in_			~ 97.29%	Mid-point
^14^	11		2	V_in_/2	1	9	11 V_in_			96.8%	Inherent
^16^	15		4	V_in_/4	6	3	6 V_in_		No	93.3%	–
^17^	16		6	V_in_	–	2	20 V_in_		Yes	NA	Mid-point
Proposed	9		8	V_in_/2	2	2	5.5V_in_			97.7%	Mid-point

TBV total blocking voltage of switches, MBV switches number of switches with maximum blocking voltage, NA not addressed.

## Power loss analysis of the proposed topology

The losses in the power components occur due to non-idealities present with them. Three components compose the multilevel ANPC inverters’ power losses are the switching losses, conduction losses of the power semiconductor devices, and ripple losses of the capacitors.

16 $$P_{loss} = P_{c} + P_{sw} + {\text{P}}_{ripple} ,$$

where *P*_*loss*_ denotes the total power loss of the MLI with *P*_*c*_, *P*_*sw*_, and *P*_*ripple*_ represents the switches losses, conduction losses and ripple losses, respectively. As a consequence of intrinsic delays in the switching of semiconductor components, during each switching transition overlaps between voltage and current leading to loss of switching which is calculated as

17 $$P_{sw} = \left[ {\sum\limits_{switches}^{{}} {\,\,\,\sum\limits_{{within\,\,{\raise0.7ex\hbox{$1$} \!\mathord{\left/ {\vphantom {1 {f_{o} }}}\right.\kern-\nulldelimiterspace} \!\lower0.7ex\hbox{${f_{o} }$}}}}^{{}} {\left( {\frac{{V_{on} \times I_{on} \times T_{on} }}{6} + \frac{{V_{off} \times I_{off} \times T_{off} }}{6}} \right)} } } \right] \times f_{f} ,$$

where, *V*_*on*_ is pre-ON state voltage across the power switch. *I*_*on*_ is the current which flows through the power switch after the ON condition. *T*_*on*_ is the period of transition in the ON state. *V*_*off*_, *I*_*off*_, and *T*_*off*_ are the voltage across the power switch, current that flows through a power switch before the transition to OFF state and transition period of the OFF state, respectively. *f*_*f*_ is the frequency of the fundamental output voltage. Conduction losses are power losses occurred due to the internal resistance offered by the switch during the conduction mode and is given as

18 $$P_{c} = \sum\limits_{all\,\,switches} {I_{sw}^{2} \times R_{on} } ,$$

where *I*_*sw*_ is the amount of current through a switch with an internal resistance of *R*_*on*_.

Aside from power losses in the switch, the ripple losses, which occur due to the charging and discharge of the capacitors, are another major contributor to the overall power loss of the MLI. When the parallel capacitor to the dc source is charged, the charging current flows through the capacitor, and because of the difference in voltage between the input source and the capacitor voltage, the ripple voltage ΔV_C_ causes the power loss. The ripple power loss of a capacitor can be calculated as

19 $$P_{ripple} = \sum\limits_{all\,\,capacitors} {C \times \Delta V_{C} \times f_{f} } .$$

**Figure 5:**
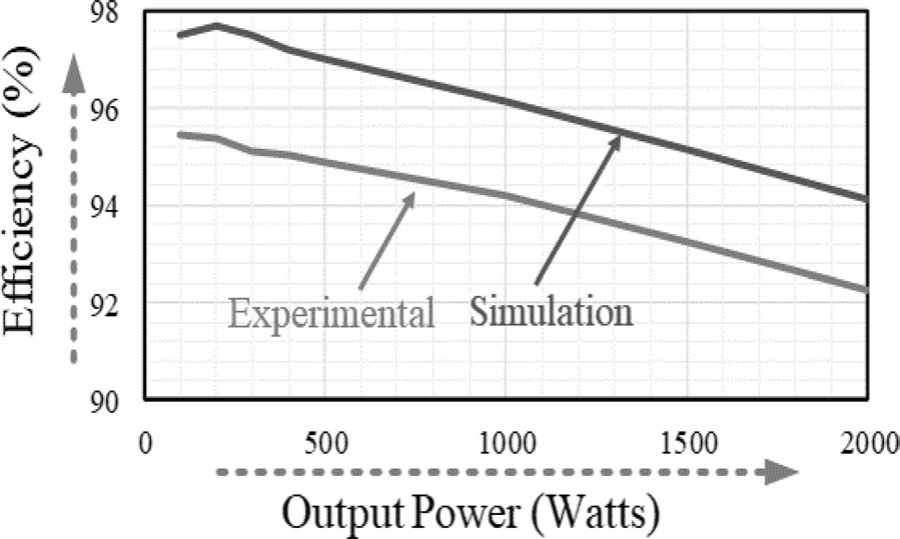
Efficiency curves of the proposed topology.

Modeling the semiconductor devices in PLECS software has led to the power load distribution for the proposed topology. The efficiency of the proposed topology against the output power has been shown in Fig. 5. The proposed topology’s maximum efficiency was measured at 200 W output power as 97.7%. The efficiency of the proposed topology is 94.1% at the output power of 2 kW. Even with higher output power, the proposed topology gives better efficiency, making it suitable for higher power applications.

**Figure 6:**
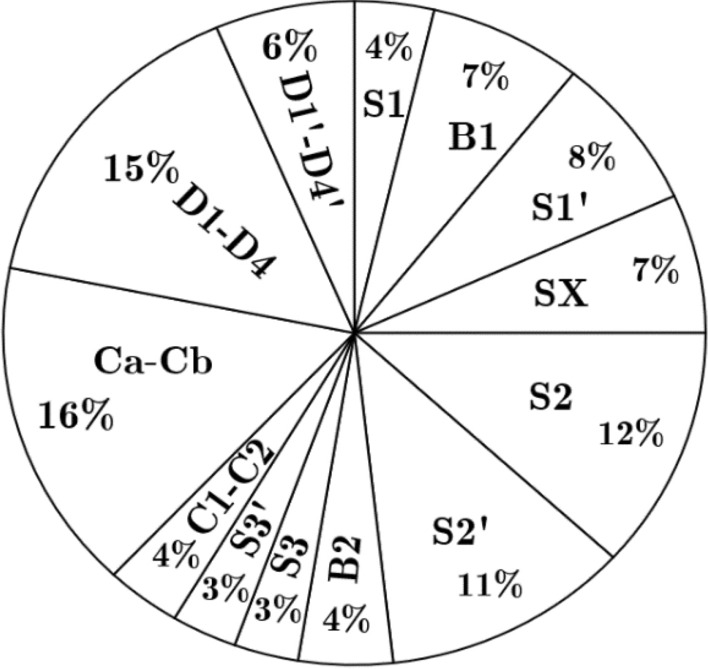
Power loss distribution.

Table 4 shows the power loss of all switches and capacitors, together with the efficiency of the proposed topology with different loading combinations. Table 4 also shows the switching power loss (P_sw_) and conduction power loss (P_c_) of the devices with a ripple power loss of the capacitors. The maximum power loss occurs to the switch pair (S_2_, S_2_′) as these switches have to carry the charging current of the capacitors C_a_ and C_b_. During charging the FCs, the charging current will be higher, leading to major losses in the devices and components. In Fig. 6, the dc-link capacitors have low power loss, but the FC losses are high (~ 16%), and the diodes presented in the mid-point of the dc-link capacitor also produce more losses because of the FC charging current. The bidirectional switch (B_1_) can be replaced with two switches that may reduce the losses and increase efficiency. Nevertheless, the switch count and driver circuit will be added extra, leading to an increase in the inverter’s cost. The sources of losses are switching the device and conduction loss during the ON time which is clearly cleared discussed in above section. Further, the other losses are: (i) DC link capacitor losses: These losses are mostly associate with capacitor voltage ripple and ESR value of the capacitors. Here, the ESR value of the capacitor is fixed by the manufacturer. (ii) Floating capacitor losses: The FC losses are high due to high charging current flowing in the charging loop. So, the FC capacitor leads to higher losses. However, in all the self-balanced switched capacitor topologies experiencing this loss.

**Table 4:** Power loss distribution of the proposed topology.

Power loss of	Output power (W)								
	100Ω + 50 mH			50Ω + 100 mH			10Ω + 100 mH		
	Psw	Pc	Ploss	Psw	Pc	Ploss	Psw	Pc	Ploss
Switch S_1_	0.6075	0.1219	0.7294	2.8914	0.1030	2.9944	1.2983	0.0185	1.3168
Switch B_1_	1.4077	0.0014	1.4091	2.6635	0.0035	2.667	0.6502	0.0003	0.6505
Switch S_1_′	1.3847	0.1117	1.4964	2.6001	0.1592	2.7593	1.1675	0.0389	1.2064
Switch S_*x*_	1.1172	0.2316	1.3488	2.1838	0.2316	2.4154	1.2343	0.1915	1.4258
Switch S_2_	2.3160	0.0407	2.3560	4.1637	0.0538	4.2175	1.5402	0.0883	1.6285
Switch S_2_′	2.2384	0.0388	2.2772	4.1613	0.0414	4.2027	1.5321	0.0736	1.6057
Switch B_2_	0.5490	0.3152	0.8642	1.2362	0.3297	1.5659	1.4434	0.3359	1.7793
Switch S_3_	0.4493	0.1427	0.592	0.9414	0.1446	1.086	0.7699	0.1070	0.8769
Switch S_3_′	0.4481	0.1545	0.6026	0.9356	0.1546	1.0902	0.7549	0.1230	0.8779
Capacitor C_1_/C_2_	0.6908			1.7347			0.2981		
Capacitor C_a_/C_b_	3.2147			8.0664			0.6308		
Didoes of switch B_1_	3.0782			5.7540			3.2717		
Didoes of switch B_2_	1.2792			2.8041			1.4694		
Total losses (W)	20.9518			48.9956			15.5274		
Efficiency (%)	97.7			95.67			94.93

**Figure 7:**
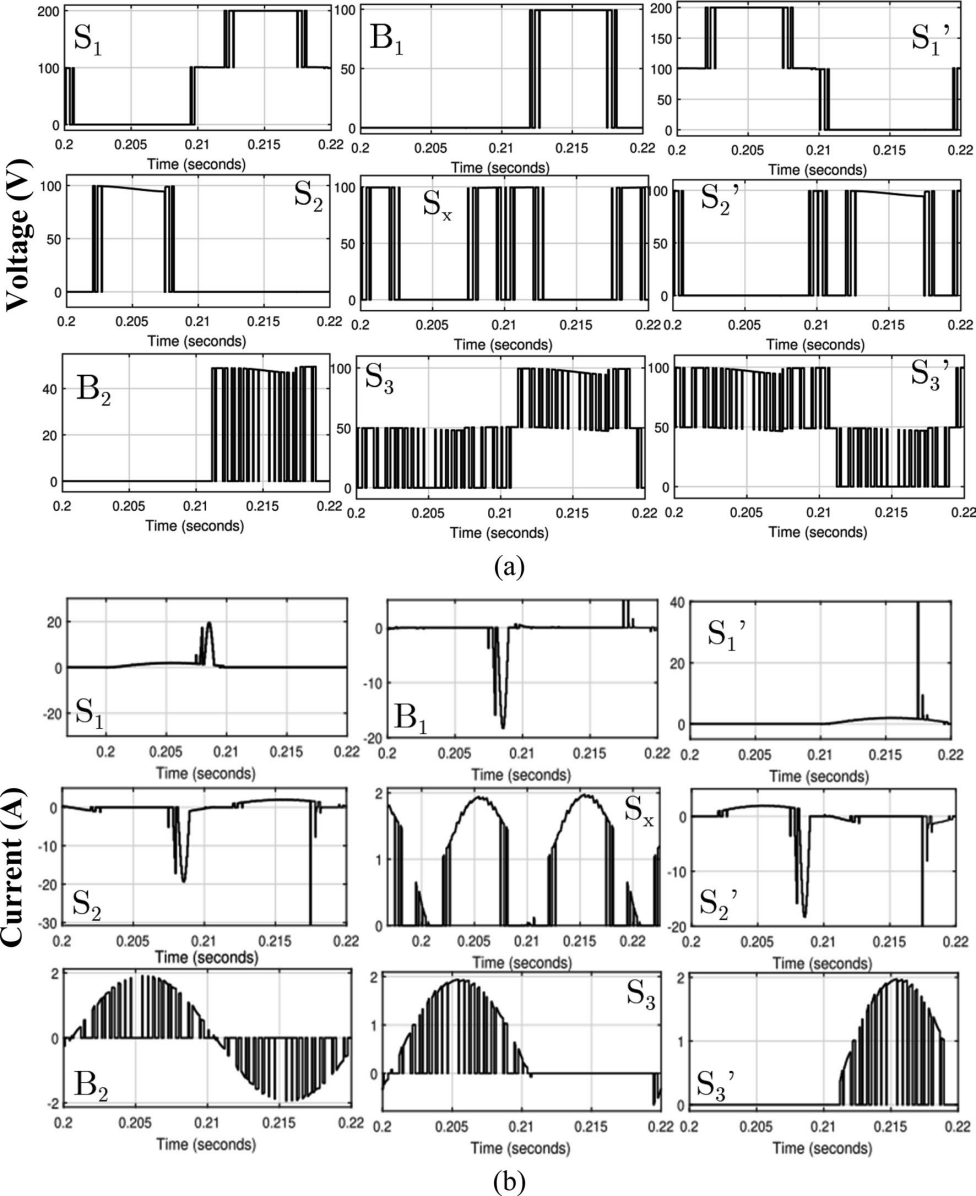
One cycle switching period waveform (**a**) for voltage and (**b**) for current.

The voltage and current across each switch for one switching period is given in Fig. 7a,b. It is confirming that the maximum blocking voltage on switch is equal to the V_in_ and the maximum current has occurred on the charging path devices.

## Experimental results

**Figure 8:**
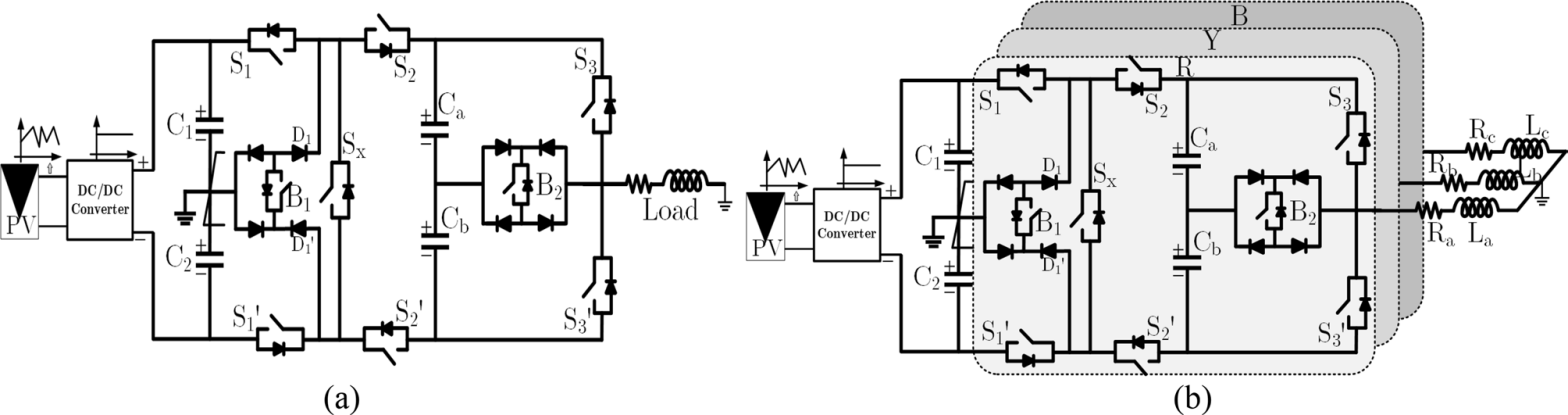
Circuit diagram of proposed 9L ANPC Topology for (**a**) single phase system and (**b**) three phase system.

**Figure 9:**
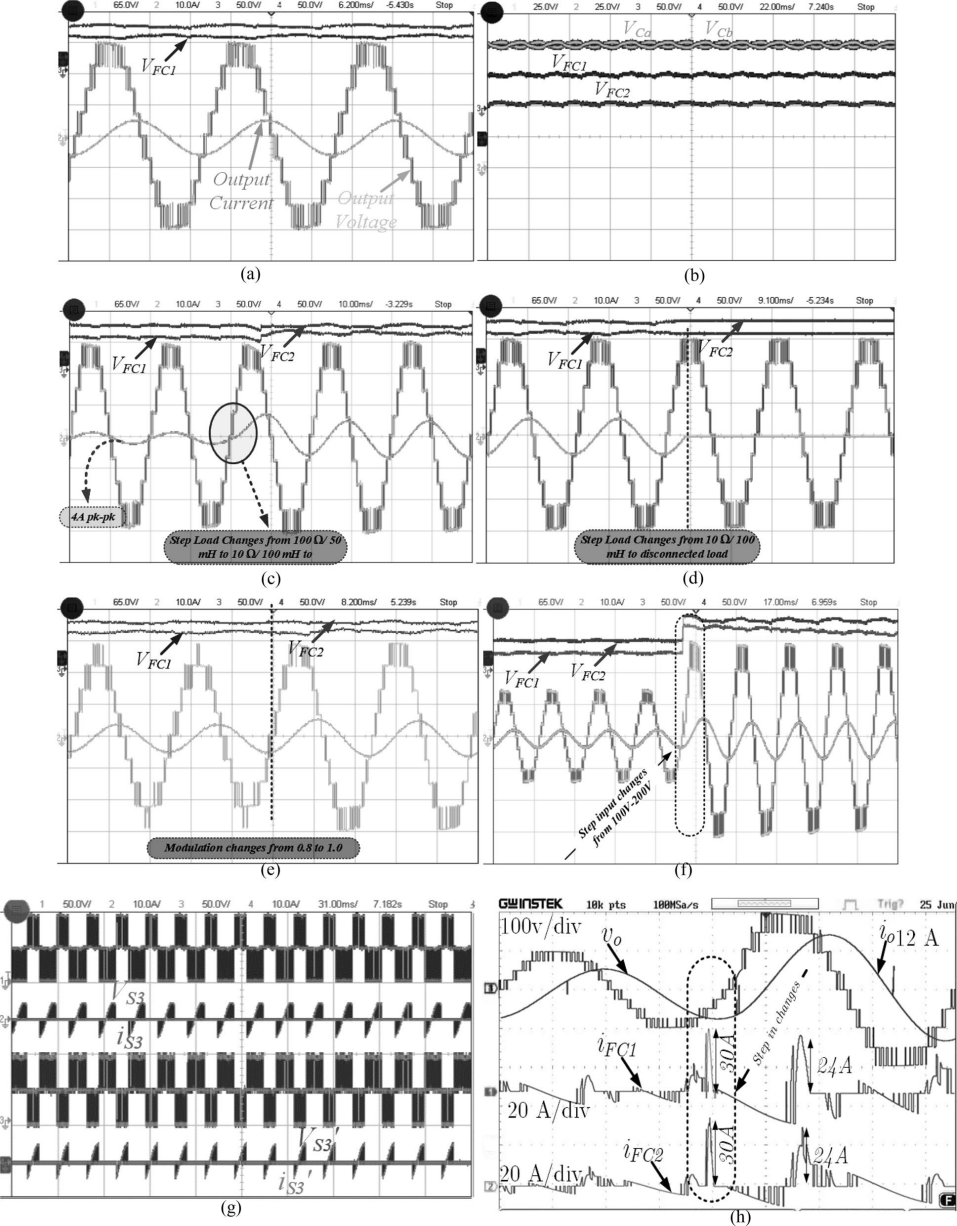
various experimental results of proposed 9L ANPC topology (**a**) output voltage and current for 10 Ω, 100 mH, (**b**) FC and dc-link capacitor voltages, (**c**) load changes from 100 Ω, 50 mH to 10 Ω, 100 mH, (**d**) load changes from 10 Ω, 100 mH to disconnected loads, (**e**) modulation index variation from 0.8 to 1.0, (**f**) the step input voltage changes from 100 to 200 V, (**g**) voltage and current of switch S_3_/S_3_′ and (**h**) floating capacitor currents during step input voltage changes.

The performance of the proposed 9L inverter is tested and verified in prototype hardware setup. The circuit diagram of proposed 9L inverter with PV applications for single and three phase system is shown in Fig. 8a,b. In hardware, the Xilinx Spartan 6 digital controller is used. The list of experimental parameters values is given in Table 5. In this V_in_ is chosen as 200 V and output voltage is 200 V with unity gain. C_1_ and C_2_ capacitors are chosen as 1700 µF with low voltage ripple of 2% but in switched cell capacitors values are selected as 2700 µF based on the ripple voltage and switching frequency of the inverter as given in Eq. (15) and Δ*V*_*C*_ is the ripple voltage of capacitor *C*_*a*_ and *C*_*b*_, which range between 0.05 to 0.1 i.e., 5% to 10% variation and the f_sw_ is switching frequency. The recommended modulation scheme is verified in experiments for a switching frequency of 2.5 kHz. Further to validate the proposed system for real-time applications, the prototype hardware model is fabricated. In hardware setup, the Semikron SKM75GB063D IGBT 600 V/75 A and TLP-250A gate driver circuits are used. The dead time of 4 µs is provided by using RC network. RL load is varied in the order of low–high-low to measure the adaptability of proposed inverter with respect to dynamic behavior and modulation under sudden load conditions as shown in Fig. 9a–g. In Fig. 9a the output voltage (M = 0.95 to 1.0) and current waveform for 10 Ω + 100 mH is presented with worst case of power factor is 0.3 and the simultaneously the dc-link capacitor and FC voltages are presented in Fig. 9b. Further, the load changes from 100 Ω, 50 mH to 10 Ω, 100 mH to confirm the suitability of proposed topology for any load variations as presented in Fig. 9c and load to no-load is shown in Fig. 9d. However, the load variations are limited based on the FC value. The maximum current through the switch is 6.0 A and the maximum blocking voltage is 200 V on S_1_ and S_1_′ switches.

**Table 5:** System parameter values.

Parameters	Experimental value	Parameters	Experimental values
DC input voltage (V_in_)	200 V	Load RL value	10 Ω, 100 mH
**Capacitor ratings**			
C_1_ and C_2_	100 V, 1700 µF		100 Ω, 50 mH
C_a_ and C_b_	50 V, 2700 µF	Digital controller	Spartan 6
**Output voltage (V**_**out**_**)**			
V_pk-pk_	195–200 V	Switching frequency	2.5 kHz
V_rms_	141.36 V	Voltage THD	12.6%
**Output current (i**_**L**_**)**			
I_pk-pk_	6.0 A and 1.9 A	Power factor (cos Ø)	0.3, 0.99
I_rms_	4.24 A, 1.34 A	Fundamental frequency	50 Hz
Output power (W)	1210.7 W, 380.0 W (with p	Output power (W) (with pf)	179.8 W, 187.52 W

**Figure 10:**
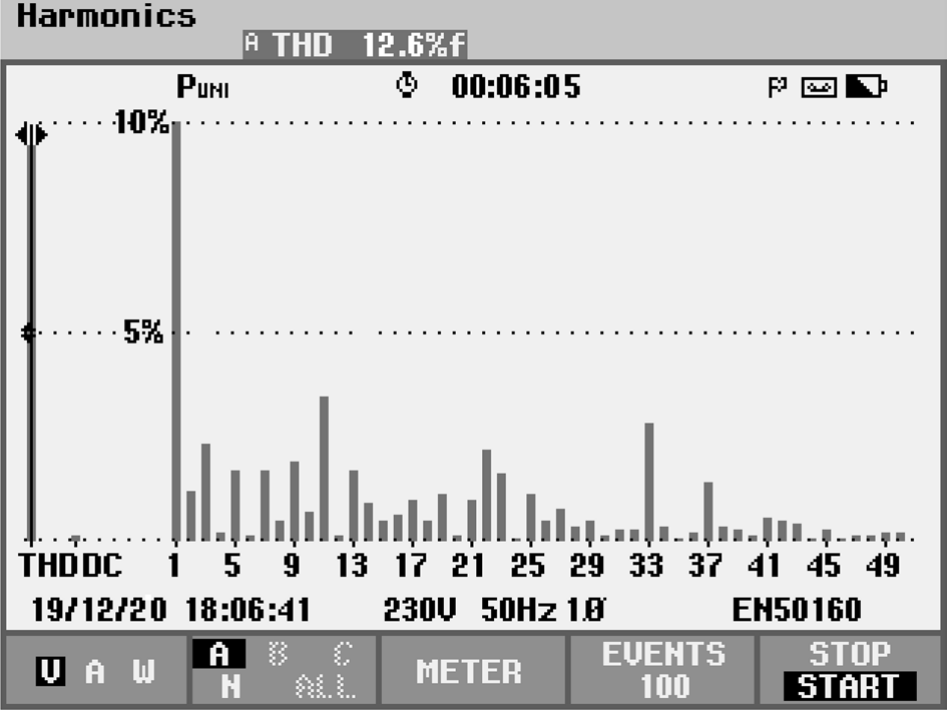
Experimental voltage THD spectrum.

**Figure 11:**
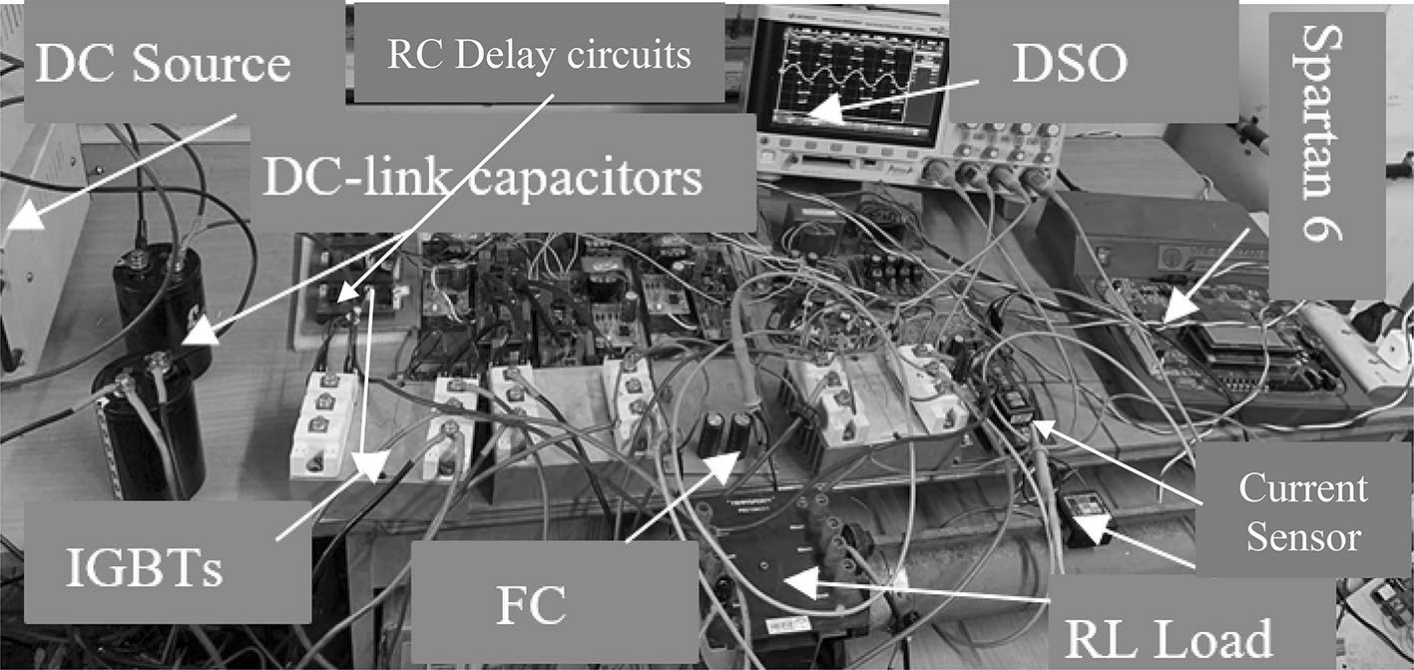
Prototype model of proposed 9L inverter.

Another dynamic variation is modulation index changes from 0.8 to 1.0, and the V_in_ changes from 100 to 200 V, as shown in Fig. 9f. In switched capacitor topology, the inrush current is another problem during the parallel connection of FC and input dc source. Due to inrush current need high current rated switches. In order to suppress the inrush current, the inductor is used in the loop, see the switch current and voltage in Fig. 9g. Further, during the step input voltage changes the capacitors charging current is increasing suddenly and it reach to ~ 30A as shown in Fig. 9h. The experimental output power is 1210.7 W for high inductive load value and 380.4 W for highly resistive load with the efficiency of 94.4% and 97.7%, respectively. The proposed inverter operates less power loss and less costly due to the low number of power components and voltage rating on the switches. Further, the voltage THD of the proposed modulation scheme is compared with the conventional phase disposition (PD), phase opposite disposition (POD) and alternate POD, and parabola modulation scheme for different modulation index (M) as listed in Table 6. The proposed modulation scheme generates THD of 12.6% in experimental for switching frequency 2.5 kHz as shown in Fig. 10. The photograph of the experimental setup for the proposed topology is given Fig. 11. The details of each components and sensors are given in Table 7.

**Table 6:** THD comparison of various modulation techniques for switching frequency of 2.5 kHz.

Modulation techniques	ma = 1.0		ma = 0.8		ma = 0.6	
	V_rms_	THD%	V_rms_	THD%	V_rms_	THD%
Sawtooth PWM	137.21	14.32	110.9	17.24	84.27	25.44
**Sinusoidal PWM**						
PD	137.65	13.66	111.2	16.99	85.34	24.36
POD	138.22	13.46	111.6	16.81	86.13	24.30
APOD	139.79	13.78	112.7	16.90	86.55	24.12
^12^	141.13	12.52	113.2	15.66	86.46	22.61
Proposed	141.74	12.04	113.6	15.23	86.42	21.33

**Table 7:** Experimental components details and rating.

Components	Model number	Voltage/current rating
DSO	X3034T/Keysight	350 MHz/4Chennal
IGBT	Semikron SKM75GB063D	600 V/75A
Dead time	RC delay circuit	3 µs
DC-link capacitor	36DX172G100AB	100 V/1700 µF
Floating capacitor	LGU1H272MELA	50 V/2700 µF
Current sensor with signal conditioning	LA 55P/715165	55 A

## Conclusion

In this paper, a 9L-ANPC type topology and its operation have been presented. The proposed topology gain is equal to the V_in_, where 200 V is applied as input and 200 V is obtained at the output. The number of switch count is reduced with reduced FC voltage rating. The proposed topology is experimentally verified, and results are presented. The proposed topology is tested with a high inductive load value of 10 Ω/100 mH, which is approximately 0.3 power factor, and the proposed topology can generate the output voltage with 9L. Further, the loss values and power loss distribution for 100 Ω + 50 mH are presented, and 97.7% efficiency is achieved. The experimental results concluded that the proposed topology has self-voltage balancing and voltage boosting ability. Further, this topology is suitable for PV applications.

## Abbreviations

NPC: Neutral point clamped
MLI: Multilevel inverter
V_in_: Input voltage
V_out_, V_o_: Output voltage
M: Modulation index
MBV: Maximum blocking voltage
TBV: Total blocking voltage
Ts: Carrier signal time interval
dq: Change of amplitude of carrier signals
ΔQ: Change of charge
θ_1_…. θ_4_: Switching angle
A_mn,_ B_mn_: Coefficients of Fourier expansion
A_oo_, B_oo_: DC offset component of the pulse width-modulated waveform
V_Carr_: Carrier signal
V_ref_: Reference signals of fundamental frequency
f_f_: Fundamental frequency
